# Retraction Note: Linc-ROR facilitates progression and angiogenesis of hepatocellular carcinoma by modulating DEPDC1 expression

**DOI:** 10.1038/s41419-025-07669-y

**Published:** 2025-04-22

**Authors:** Chuan Tian, Mubalake Abudoureyimu, Xinrong Lin, Xiaoyuan Chu, Rui Wang

**Affiliations:** Department of Medical Oncology, Affiliated Jinling Hospital, Medical School of Nanjing University, Nanjing, China

Retraction to: *Cell Death and Disease* 10.1038/s41419-021-04303-5, published online 05 November 2021

The Editors-in-Chief have retracted this article at the authors’ request. After publication, concerns were raised regarding potential image overlap in Figs. 4 and 6, specifically:

- Fig. 4b Hep3B DEPDC1 image appears to overlap with Fig. 6b Hep3B Lv-ROR;
- Fig. 4d HepG2 vec+miR-NC appears to overlap with Fig. 4h Lv-shDEPDC1;
- Fig. 4h Hep3B Lv-shDEPDC1 appears to originate from the same sample as Fig. 6d Hep3b Lv-ROR+Lv-shDEPDC1;
- Fig. 4f Hep3B Lv-shDEPDC1 appears to overlap with Fig. 6f HepG2 Lc-shROR;
- Fig. 6f Hep3b Lv-shROR+DEPDC1 appears to overlap with Fig. 6b HepG2 LV-NC+Lv-shNC and Fig. 6f Hep3B Lv-shROR.

Additionally, the authors have used the L02 cell line, which has been reported to be contaminated with HeLa cervical cancer cells, as a model of normal liver cells. The HepG2 cell line used in the in vitro and in vivo experiments has been reported to be misclassified and originate from hepatoblastoma rather than hepatocellular carcinoma.

The authors are re-analysing their data and intend to submit a new manuscript for peer review in due course.

Chuan Tian and Rui Wang agree to this retraction, but not the wording of this retraction notice. Mubalake Abudoureyimu, Xinrong Lin and Xiaoyuan Chu have not responded to any correspondence from the editor or publisher about this retraction.

